# Holstein Polarons, Rashba-Like Spin Splitting, and Ising Superconductivity in Electron-Doped MoSe_2_

**DOI:** 10.1021/acsnano.4c07805

**Published:** 2024-11-26

**Authors:** Sung Won Jung, Matthew D. Watson, Saumya Mukherjee, Daniil V. Evtushinsky, Cephise Cacho, Edoardo Martino, Helmuth Berger, Timur K. Kim

**Affiliations:** † Diamond Light Source, Harwell Science and Innovation Campus, Didcot OX11 0DE, U.K.; ‡ Department of Physics and Research Institute of Molecular Alchemy, Gyeongsang National University, Jinju 52828, Republic of Korea; § Van der Waals-Zeeman Institute, Institute of Physics, University of Amsterdam, Amsterdam 1098 XH, Netherlands; ∥ École Polytechnique Fédérale de Lausanne, Lausanne CH-1015, Switzerland

**Keywords:** transition metal dichalcogenides, surface doping, electronic structure, polarons, Ising superconductivity, ARPES

## Abstract

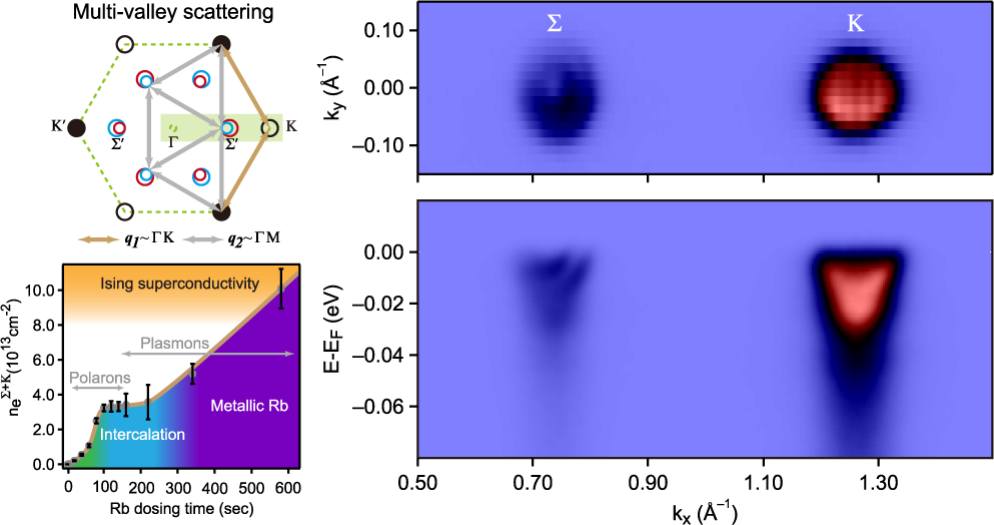

Interaction between electrons and phonons in solids is a key effect defining the physical properties of materials, such as electrical and thermal conductivity. In transition metal dichalcogenides (TMDCs), the electron–phonon coupling results in the formation of polarons, quasiparticles that manifest themselves as discrete features in the electronic spectral function. In this study, we report the formation of polarons at the alkali-dosed MoSe_2_ surface, where Rashba-like spin splitting of the conduction band states is caused by an inversion-symmetry breaking electric field. In addition, we observed a crossover from phonon-like to plasmon-like polaronic spectral features at the MoSe_2_ surface with increasing doping. Our findings support the concept of electron–phonon coupling-mediated superconductivity in electron-doped layered TMDC materials, as observed using ionic liquid gating technology. Furthermore, the discovered spin-splitting at the Fermi level could offer crucial experimental validation for theoretical models of Ising-type superconductivity in these materials.

## Introduction

Polarons are collective excitations of electrons interacting with the lattice. These quasiparticles describe an electron moving in a crystal with positively charged ions displacing from their equilibrium positions to effectively screen negative electron charges.^1,2^ This coupling between the electron and lattice vibrations (phonon cloud) lowers the electron mobility and increases its effective mass.

The resulting polaronic bound state can be described by Fröhlich or Holstein Hamiltonians. The Fröhlich model describes the interaction of electrons with phonons, which leads to the formation of large polarons with radii much larger than the lattice constant. The Holstein model, on the other hand, describes the short-range interaction with phonons, which typically leads to the formation of small polarons, whose radii are of the same order of magnitude as the lattice constant.^3^

Formation of polarons due to a momentum-dependent electron–phonon coupling leads to new features in the electronic spectral function at specific electron valleys, e.g., the emergence of discrete energy levels and discontinuities in the density of states.^4^ In a recent study of alkali-metal dosing on MoS_2_ surface such discontinuities in the density of states have been experimentally observed at the *K* points of the Brillouin zone using angle-resolved photoemission spectroscopy (ARPES).^5^ Understanding the changing nature of the quasiparticles as a function of doping is an important prerequisite for understanding the onset of superconductivity observed in highly electron-doped MoS2 under liquid ionic gating.^6,7^

**Figure 1:**
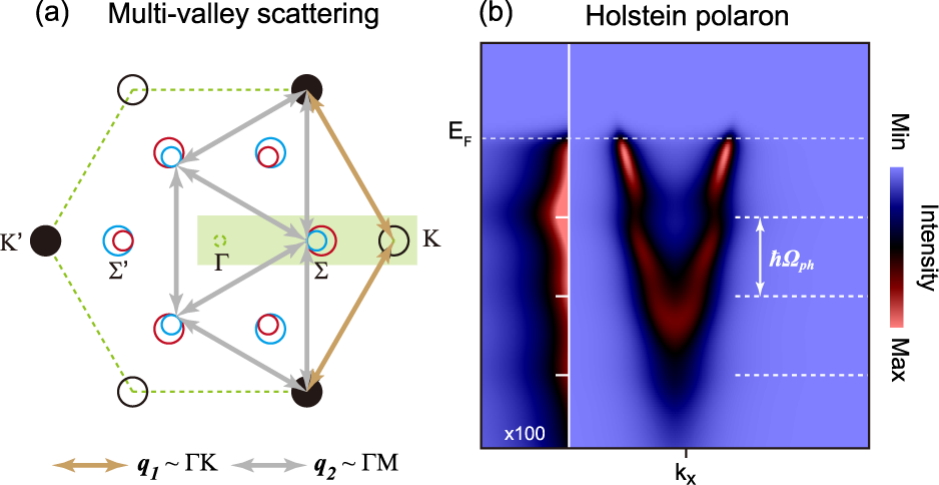
Phonon-mediated polarons in electron-doped MoSe_2_: (a) multivalley scattering of conduction electrons in the first Brillouin zone of MoSe_2_; (b) electron-like parabolic band at K-valley with Holstein polarons simulated by momentum-average model.^9^

Theoretical considerations point to two key features in the case of multivalley electron-doped TDMCs. First, polaron modes should be observed around both K and Σ valleys in MoS_2_.^8^Figure 1a shows the multivalley scattering between Σ and K valleys in the Brillouin zone. The electron scattering vectors *q*_1_ and *q*_2_ correspond to either Γ–K or Γ–M phonon wave vectors. The dominant contribution to electron–phonon coupling arises from the highest-energy acoustic phonon mode. This behavior has been demonstrated both experimentally^10^ and theoretically^11^ for MoS_2_. As in the case of MoS_2_,^11,12^ for MoSe_2_, the acoustic phonon modes have essentially flat dispersion over an extended region of the *k*-space between the M and K points.^13^ Thus, despite their acoustic nature, these phonon modes with characteristic frequency Ω_ph_ define the energy scale for electron–phonon coupling and the corresponding polaron formation.^12,13^ However, only K valley Holstein polarons (Figure 1b) have been observed in the ARPES study of the Rb-dosed MoS_2_ surface.^5^ Second, the spin–orbit coupling (SOC) should induce the splitting of the electron bands at Σ and Σ′ valleys due to inversion symmetry breaking on the surface with alkali-metal dosing. However, in all previous ARPES studies of alkali-dosed semiconducting TMDCs, the spin-split features at Σ valley have not been resolved.^5,14−23^

The isostructural MoSe_2_ compound is the perfect model system for probing the multivalley physics induced by electron doping. Density functional theory (DFT) calculations show that, in contrast to MoS_2_, the conduction band minimum (CBM) in bulk MoSe_2_ is at the Σ, rather than at K valley (see Figure S1). For alkali-metal dosing on the MoSe_2_ surface, one expects to have larger SOC band splitting than in the case of MoS_2_. The accessibility of the Σ valley, as well as the K valley expected at higher doping,^20,21^ makes MoSe_2_ the best candidate to study the physics of multivalley polaron modes using high-resolution ARPES.

## Results and Discussion

### Evolution of the Electronic Band Structure with Dosing

**Figure 2:**
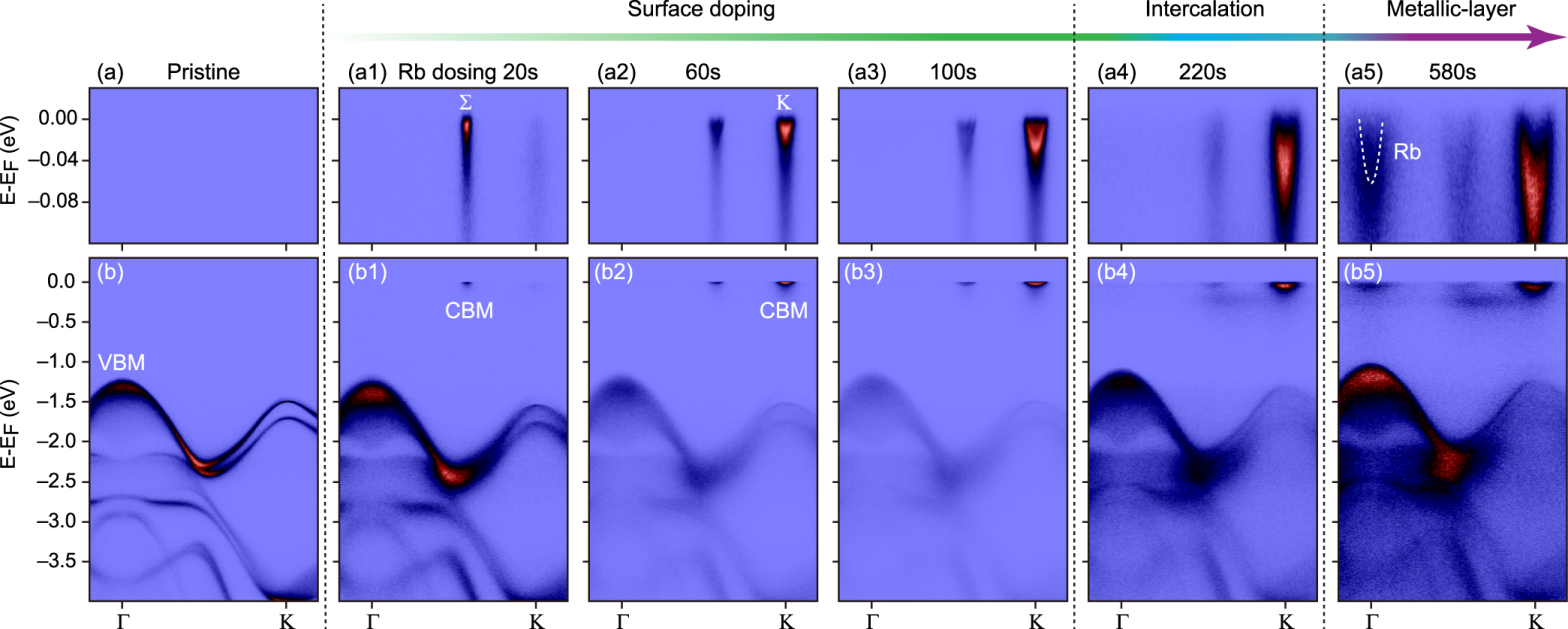
Evolution of the MoSe_2_ electronic structure with Rb dosing: (a–a5) ARPES spectra of Γ-K conduction band dispersions measured near the Fermi energy for cumulative dosing time 0, 20, 60, 100, 220, 580s. (b–b5) Corresponding valence band spectra over a wide energy range.

In our ARPES data shown in Figure 2, the spectral intensity appears at the Fermi level, marking the occupation of the conduction band and corresponding semiconductor to conductor transition with Rb dosing on the MoSe_2_ surface. After the initial dosing, the maximum spectral weight at the Fermi level was observed around Σ valley, showing an indirect band gap  ∼ 1.4 eV. However, with increased amount of dosed Rb, the relative intensity of the Σ valley decreased, whereas the intensity of K valley increased. Correspondingly, it appears that the conduction band minimum shifts from the Σ to K valley, as in the case of monolayer MoSe_2_ (see Figure 2a1–a3). Within the observed energy broadening, the band minima at Σ and K are very close (see Figure S5), incidentally making electron-doped MoSe_2_ a perfect system for studying multivalley physics. Simultaneously with the shift of the CBM intensity, the intensity of the valence band compared to the conduction band decreases (Figure 2b1–b3). This first crossover, at around 40 s of Rb dosing, marks the transition from the electron-doped bulk to the more “monolayer-like” doped surface conduction band structure.^15,17,21,22^

The accumulation of alkali atoms on the surface and increased electron doping of the top layer causes a corresponding increase of the electric field and results in the amplification of the inversion symmetry breaking at the surface. At the dosing above this first crossover, the measured electronic structure is a superposition of the bands from both the bulk material and the doped surface. This observation is supported by our Se 3d core level and K-valley valence band data (see Figures S4 and S5 correspondingly).

At Rb dosing times above 120 s, the relative intensity of the valence bands increases due to the interlayer intercalation of Rb atoms.^15^ At the same time, the energy difference between the bulk and surface Se 3d core levels or “effective chemical shift” saturates and does not increase with the Rb deposition time (see Figure S4). This corresponds to the second crossover from surface doping to interlayer intercalation regime. For dosing times above 340 s, we started to observe a buildup of the photoemission intensity at the Fermi level around the Γ-point as seen in Figure 2a5. This corresponds to the formation of metallic Rb states on the MoSe_2_ surface^21^ and the third crossover from the interlayer intercalation regime to the growth of an ordered alkali metal layer on the surface.

### Holstein Polarons in “Surface Doping” Regime

**Figure 3:**
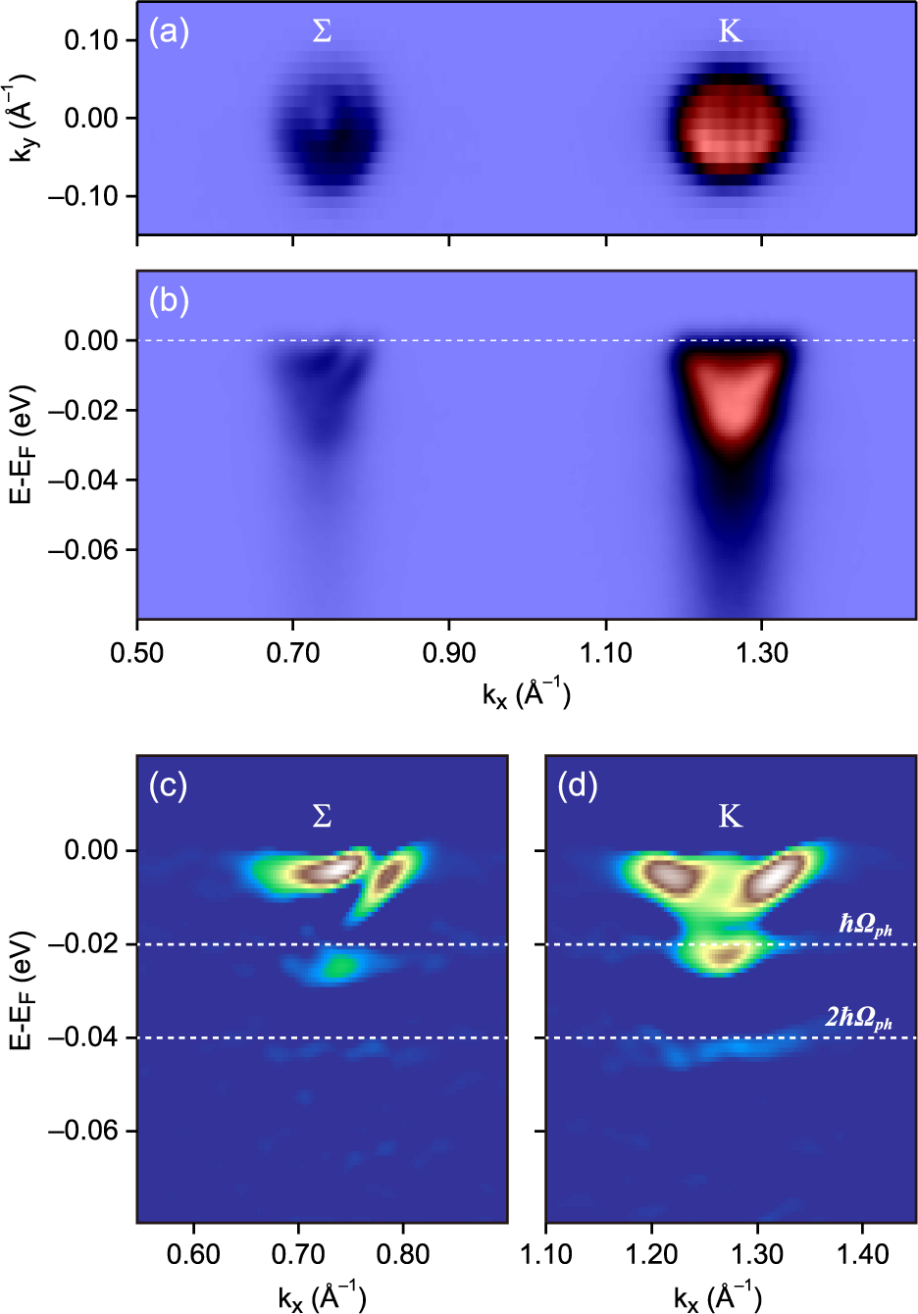
Formation of polarons in the electron-doped MoSe_2_: (a) Fermi surface and (b) Σ–K band dispersion measured after 120 s Rb dosing; corresponding second derivative of ARPES intensity at the Σ (c) and K (d) valleys.

The most intriguing effects in the electronic structure are observed in the “surface doping” regime, corresponding to the dosing times between the first and second crossover. Figure 3a,b shows the electronic band structure of MoSe_2_ at the Fermi level after 120 s of Rb dosing on the surface. The measured Fermi surface shows two electron-like pockets at Σ valley and a single electron-like pocket at the K valley. The second-derivative images of Σ and K valleys in Figure 3c,d both show well-defined discontinuities of the spectral function, separated by 20 meV. Moreover, the photoemission spectra integrated withing a selected momentum range between Σ and K valleys in Figure 5a, show discrete nondispersing energy levels with the same energy separation (dashed vertical black lines in Figure 5b).

The energy of these spectral features is consistent with the calculated acoustic phonon mode, and their nondispersing character in an extended momentum space all point toward the formation of small-range polarons. Therefore, for the electron-doped MoSe_2_, Holstein-type polarons are observed at both the K and Σ valleys.

### Spin Splitting at the Σ Valley in “Surface Doping” Regime

**Figure 4:**
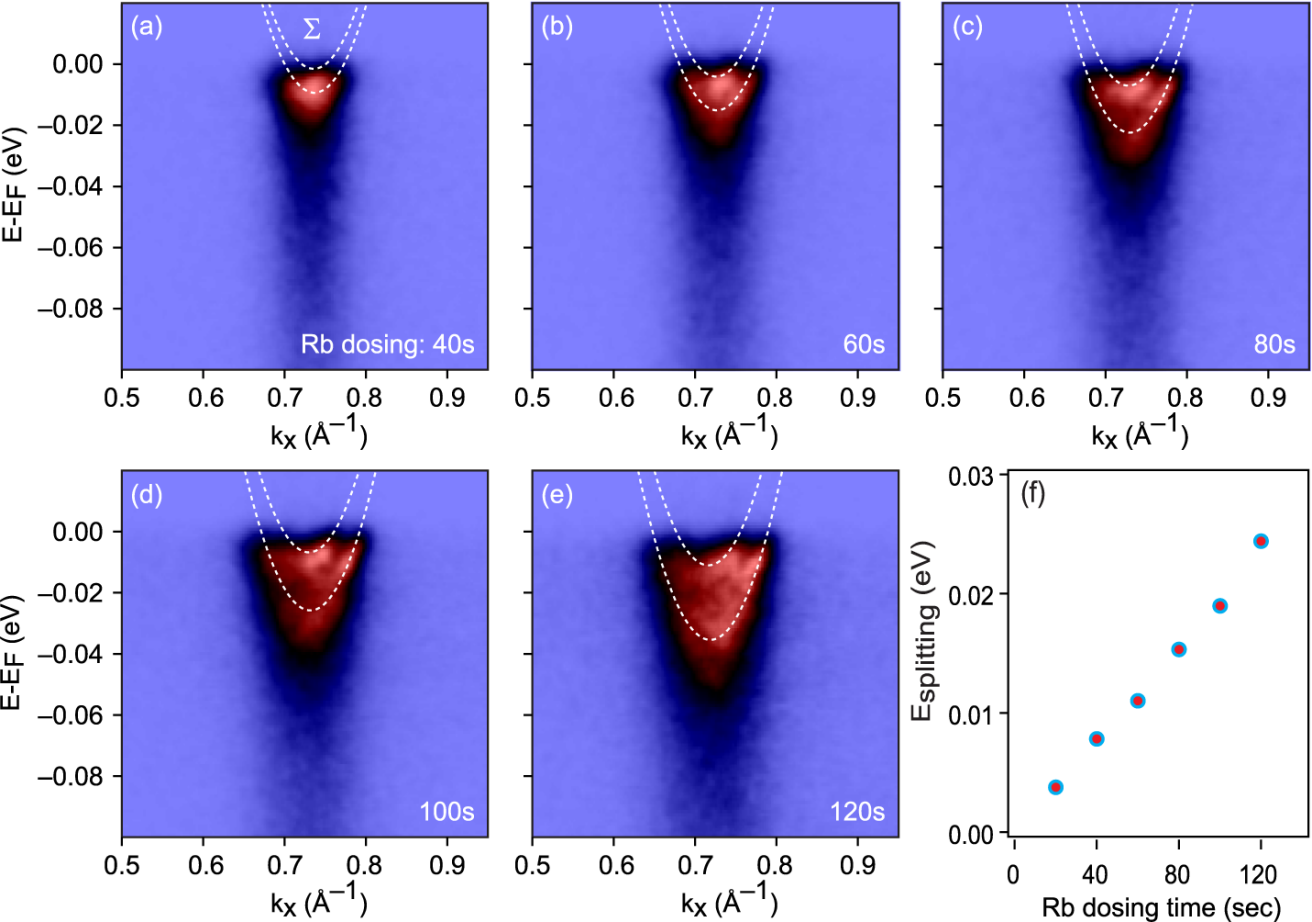
Evolution of the spin–orbit coupling with Rb dosing: (a–e) band dispersion at the Σ valley with Rb dosing. White dotted lines are fits with Rashba-like model, with Γ been time-reversal invariant momentum (TRIM) point. (f) Extracted spin-split energy at Σ valley.

In addition to the polarons at the spin-degenerate K valley previously observed in MoS_2_,^5^ we have also identified polarons at the spin-polarized Σ valleys. The spin–orbit splitting of the electron band dispersion at the Σ valley is apparent in the ARPES data measured along the Γ–K direction. This result agrees with theoretical predictions of lifting the spin degeneracy under electron doping and much larger spin–orbit splitting at Σ compared to K due to the distinct orbital character of conduction bands at these valleys.^24−26^Figure 4 shows the band dispersions at the Σ valley together with the Rashba-like model fit constrained by the band positions from the corresponding momentum distribution curves (MDCs) at the Fermi level. The increase in the Fermi surface size indicates the gradual electron doping of MoSe_2_ with Rb dosing. From the same data, one could see that the magnitude of band splitting is also increasing with electron doping. This Rashba-like spin-splitting of the bands comes from inversion symmetry breaking due to the electric field created by electron charge transfer from dosed alkali atoms at the surface. Such inversion symmetry breaking on the surface has been seen in the valence band of semiconducting^21,22^ as well as in the conduction band of semimetallic^25^ alkali-dosed TMDC materials. Figure 4f shows how the magnitude of the observed splitting consistently and monotonically increased as the surface charge increased. This observation aligns perfectly with our understanding that band splitting is Rashba-like and arises from the combination of spin–orbit coupling with the increasing inversion symmetry-breaking electric field at the surface. Moreover, the constancy of intensity for the two components with dosing rules out the case of a superposition of the surface and subsurface as a possible origin of the observed splitting.

### Plasmonic Flat States in Intercalation Regime

**Figure 5:**
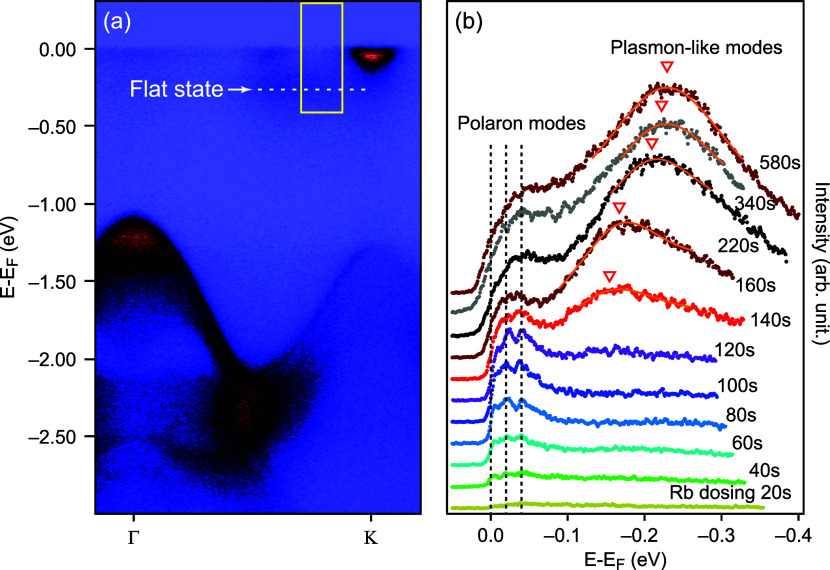
Crossover from polarons to plasmons in the MoSe_2_ electronic structure with Rb dosing: (a) Wide energy range Γ–K band dispersion measured after 340 s of Rb dosing. A white arrow indicates a flat state between Σ and K valleys; (b) Integrated Energy Distribution Curves (EDCs) between Σ and K valleys (yellow box in (a)).

At dosing times above 340 s, one observes a formation of the flat states in the vicinity of the CBM with much higher binding energies than for Holstein polaronic modes (white arrow at Figure 5a). To emphasize the difference between these new flat states and Holstein polarons, we analyzed the momentum-integrated Energy Distribution Curves (EDCs) between Σ and K valleys, as shown in Figure 5a. Figure 5b shows that indeed two different features are visible in the integrated EDCs. The characteristic binding energies of the polaronic states in the “surface doping” regime are constant and correspond to an acoustic phonon frequency of ≃20 meV, but the higher binding energies of the flat states in the “interlayer intercalation” regime significantly increase with dosing. While the binding energies of these flat states are an order of magnitude larger than those of Holstein polarons, they are also an order of magnitude smaller then the 3.4 eV bulk plasmon frequency of metallic Rb.^27^ The binding energies of the flat states as a function of the 2D electron carrier density can be fitted with the plasmon-like dispersion . The obtained dielectric constant ϵ ≃ 14.3 is comparable with the calculated in-plane dielectric constant for monolayer MoSe_2_.^28^ This suggests that these flat states may indeed be plasmonic polarons, similar to the case of EuO^29^ and anatase TiO_2_.^30^ Plasmonic polarons have been observed by ARPES in electron-doped MoS_2_,^31^ MoS_2_/TiO_2_,^32^ HfS_2_,^33^ and WS_2_.^34^

## Conclusion

**Figure 6:**
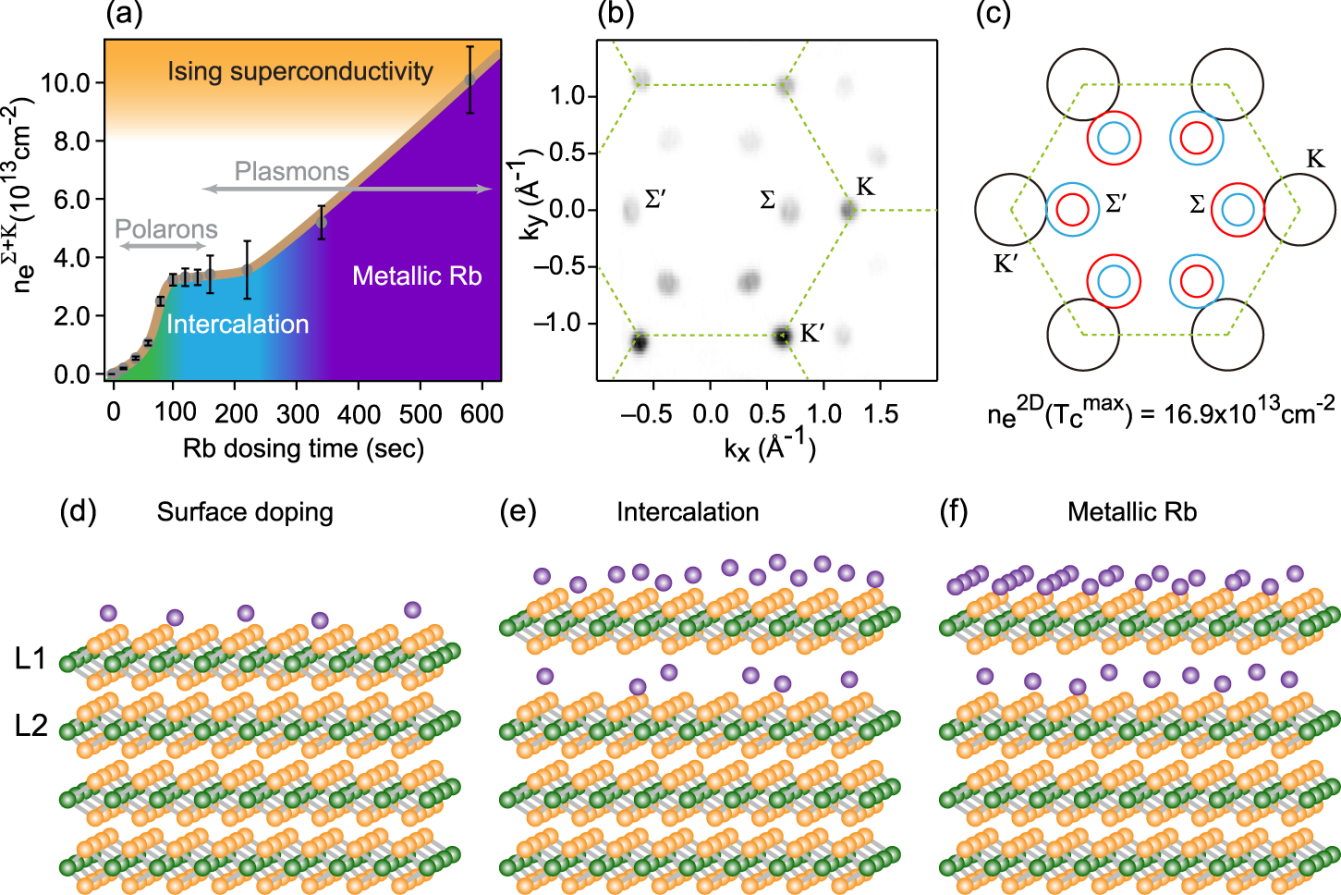
Phase diagram and Fermi surface of MoSe_2_ with electron doping: (a) 2D electron carrier density as a function of the Rb-dosing time; (b) measured Fermi surface of the electron-doped MoSe_2_ after 140 s of Rb dosing. (c) Fermi surface extrapolated for maximum *T*_c_ ∼ 7 K carrier density doping level;^35^ (d–f) Schematic of the crystal structure for surface dosing, intercalation and metallic Rb regimes. L1 and L2 note the topmost and second topmost layers, correspondingly.

By studying the electronic structure evolution of MoSe_2_ with Rb surface dosing, we have identified three different regimes: surface doping, interlayer intercalation, and ordered alkali-metal layer formation. Figure 6a shows the two-dimensional (2D) electron carrier density as a function of the alkali-metal dosing time, as calculated using Luttinger’s theorem and experimentally determined sizes of the Fermi surface. In the “surface doping” regime, we observed continuous electron doping of MoSe_2_ with a corresponding increase of the Fermi surface size and value of the Rashba-like spin-orbit splitting. In this regime, before interlayer intercalation occurs, Rb dosing on the surface is comparable to doping induced by electrostatic gating using ionic liquid. Therefore, the electronic structure on MoSe_2_ under Rb dosing in the “surface doping” regime corresponds to the electronic structure of MoSe_2_ in an ionic liquid gating device under positive gate voltage (electron doping).

The changing nature of the quasiparticles at the Fermi level due to electron–phonon coupling, as well as the evolution of the Fermi surface topology and the electronic density of states, plays a crucial role in understanding superconductivity in electron-doped TMDC semiconductors discovered using liquid gating devices.^6,7,35,36^ In the case of MoSe_2_, the superconductivity occurs at high 2D electron carrier densities ≥8 × 10^13^ cm^–2^ and maximum *T*_c_ ∼ 7 K is achieved at electron density ≃16.9 × 10^13^ cm^–2^, correspondingly.^35^

While our results show that the increasing surface electric field description is no longer applicable when we dose Rb atoms above 120 s, we could extrapolate a gradual increase of the 2D electron carrier density with the alkali metal dosing time within the “surface doping” regime up to the superconducting transition. In this case, using extrapolation from our ARPES data, we conclude that the maximum *T*_c_ doping level corresponds to the Fermi surface topology where the electron-like pocket at K valley touches the outer electron pocket at Σ valley (see Figure 6b). In another words, the maximum *T*_c_ corresponds to the Fermi level being located exactly at the conduction band saddle point between the K and Σ valleys. This can lead to a dramatic enhancement of pairing strength due to strong increase of the momentum-resolved electron–phonon coupling constant λ, like in the case of Rb-intercalated PtTe_2_.^37^ It can be assumed that with a further increase in doping, the electronic structure will undergo a Lifshitz transition, significantly reducing the density of states at the Fermi level and, correspondingly, suppressing electronic pairing. This can naturally explain the observed superconducting dome for *T*_c_ vs doping phase diagram.^6,35^

Using ionic liquid gating devices, it was found that for electric-field-induced superconductivity in MoS_2_, the observed upper critical field four–five times exceeds the Pauli limit.^7,38^ This violation of the paramagnetic limit and significant spin–orbit coupling at the Fermi level suggest a nonspin-singlet pairing that is robust against an external magnetic field. Ising pairing for the valley alternating spin-split Fermi surface has been suggested in several theoretical works for noncentrosymmetric TMDC superconductors.^39−44^ However, all these models consider basic Fermi-surface topology with spin-degenerate states in the K valleys only. At the same time, our study shows direct evidence of Rashba-like splitting of the bands at the Σ valley for such in situ electron-doped materials.

An important consequence of this discovery is that a significant part of the total Fermi surface is spin split at optimal doping. This agrees with the theoretical prediction^45^ that with antisymmetric field-effect doping, the resulting Fermi surface is dominantly formed by the pockets around Σ valleys (only ∼30% of its area is around the K valleys, as shown in Figure 6b). Thus, any realistic pairing model of Ising superconductivity in electron-doped TMDCs should include such composite spin-split multivalley Fermi surface topology and corresponding interband scattering processes.

To summarize, we observed spin-polarized Holstein-type polaronic quasiparticles on Rb-dosed MoSe_2_ surfaces. This is direct evidence of the predicted Rashba-like spin–orbit split polarons.^46^ We found that beyond the “surface doping” regime threshold, when dosed alkali atoms intercalated into the van der Waals (vdW) gap between the layers, completely different plasmon-like quasiparticles emerged. Our findings not only suggest the crucial role of polaron formation for the electronic structure of electron-doped transition-metal dichalcogenides but also provide experimental foundation for theoretical models of Ising-type superconductivity in these materials.

## Methods

High-quality single crystals of MoSe_2_ were grown by the chemical vapor transport technique.^47^ ARPES measurements were performed at the I05 beamline at the Diamond Light Source, UK.^48^ The photoelectron energy and angular distributions were analyzed with a SCIENTA R4000 hemispherical analyzer. The angular resolution was 0.2°, and the overall energy resolution was better than 5 meV. P-polarized 60 eV photons corresponding to the Γ point of the bulk Brillouin zone were used for high-resolution ARPES, and 120 eV photons for Se 3d core level measurements. Single crystal samples were cleaved and measured in ultrahigh vacuum at temperatures below 10 K and pressures below 1 × 10^–10^ mbar. Rb atoms were dosed in several sequences on a cold MoSe_2_ surface by using a commercial SAES alkali-metal dispenser. Density functional theory (DFT) calculations, including spin–orbit coupling with generalized gradient approximation (GGA), were performed using the Wien2K package.^49^ Spectral function simulations for polarons were done by using momentum average (MA) approximation^9^ with 20 meV constant phonon energy.

## Supporting Information Available

DFT calculations with spin–orbit coupling for bulk MoS_2_, bulk and monolayer MoSe_2_ (Figure S1). Photoemission data for conduction (Figure S2) and valence (Figure S3) bands together with corresponding Se 3d core levels data (Figure S4) measured for all Rb dosing sequences. Doping evolution of the band gap, conduction band minima and valence band maxima are shown in Figures S5–S7. Constant energy momentum distribution maps of MoSe_2_ after Rb dosing are plotted in Figure S8. Model of the Rashba-like splitting at Σ valley with extracted splitting are shown in Figures S9 and 10 (PDF)

## References

[ref1] Landau L. D. Electron motion in crystal lattices. Phys. Z. Sowjet 1933, 3, 664.

[ref2] Landau L.; Pekar S. Effective mass of a polaron. Zh. Eksp. Teor. Fiz 1948, 18, 419–423.

[ref3] Emin D. Polarons; Cambridge University Press, 2013.

[ref4] Franchini C.; Reticcioli M.; Setvin M.; Diebold U. Polarons in materials. Nat. Rev. Mater 2021, 6, 560–586. 10.1038/s41578-021-00289-w.

[ref5] Kang M.; Jung S. W.; Shin W. J.; Sohn Y.; Ryu S. H.; Kim T. K.; Hoesch M.; Kim K. S. Holstein polaron in a valley-degenerate two-dimensional semiconductor. Nat. Mater 2018, 17, 676–680. 10.1038/s41563-018-0092-7. 29807984

[ref6] Ye J.; Zhang Y. J.; Akashi R.; Bahramy M. S.; Arita R.; Iwasa Y. Superconducting dome in a gate-tuned band insulator. Science 2012, 338, 1193–1196. 10.1126/science.1228006. 23197529

[ref7] Lu J.; Zheliuk O.; Leermakers I.; Yuan N. F.; Zeitler U.; Law K. T.; Ye J. Evidence for two-dimensional Ising superconductivity in gated MoS_2_. Science 2015, 350, 1353–1357. 10.1126/science.aab2277. 26563134

[ref8] Garcia-Goiricelaya P.; Lafuente-Bartolome J.; Gurtubay I. G.; Eiguren A. Long-living carriers in a strong electron–phonon interacting two-dimensional doped semiconductor. Commun. Phys 2019, 2 (1), 81 10.1038/s42005-019-0182-0.

[ref9] Goodvin G. L.; Berciu M.; Sawatzky G. A. Green’s function of the Holstein polaron. Phys. Rev. B 2006, 74, 245104 10.1103/PhysRevB.74.245104.

[ref10] Zeng H.; Dai J.; Yao W.; Xiao D.; Cui X. Valley polarization in MoS_2_ monolayers by optical pumping. Nat. Nanotechnol 2012, 7, 490–493. 10.1038/nnano.2012.95. 22706701

[ref11] Kaasbjerg K.; Thygesen K. S.; Jacobsen K. W. Phonon-limited mobility in *n*-type single-layer MoS_2_ from first principles. Phys. Rev. B 2012, 85, 115317 10.1103/PhysRevB.85.115317.

[ref12] Zhao Y.; Dai Z.; Zhang C.; Lian C.; Zeng S.; Li G.; Meng S.; Ni J. Intrinsic electronic transport and thermoelectric power factor in n-type doped monolayer MoS2. New J. Phys 2018, 20, 043009 10.1088/1367-2630/aab338.

[ref13] Bae S.; Matsumoto K.; Raebiger H.; Shudo K.-I.; Kim Y.-H.; Handegård Ø. S.; Nagao T.; Kitajima M.; Sakai Y.; Zhang X.; Vajtai R.; et al. K-point longitudinal acoustic phonons are responsible for ultrafast intervalley scattering in monolayer MoSe2. Nat. Commun 2022, 13 (1), 4279 10.1038/s41467-022-32008-6. 35879336 PMC9314385

[ref14] Zhang Y.; Chang T.-R.; Zhou B.; Cui Y.-T.; Yan H.; Liu Z.; Schmitt F.; Lee J.; Moore R.; Chen Y.; et al. Direct observation of the transition from indirect to direct bandgap in atomically thin epitaxial MoSe_2_. Nat. Nanotechnol 2014, 9, 111–115. 10.1038/nnano.2013.277. 24362235

[ref15] Eknapakul T.; King P.; Asakawa M.; Buaphet P.; He R.-H.; Mo S.-K.; Takagi H.; Shen K.; Baumberger F.; Sasagawa T.; et al. Electronic structure of a quasi-freestanding MoS_2_ monolayer. Nano Lett 2014, 14, 1312–1316. 10.1021/nl4042824. 24552197

[ref16] Alidoust N.; Bian G.; Xu S.-Y.; Sankar R.; Neupane M.; Liu C.; Belopolski I.; Qu D.-X.; Denlinger J. D.; Chou F.-C.; et al. Observation of monolayer valence band spin-orbit effect and induced quantum well states in MoX_2_. Nat. Commun 2014, 5, 4673 10.1038/ncomms5673. 25146151

[ref17] Riley J. M.; Meevasana W.; Bawden L.; Asakawa M.; Takayama T.; Eknapakul T.; Kim T.; Hoesch M.; Mo S.-K.; Takagi H.; et al. Negative electronic compressibility and tunable spin splitting in WSe_2_. Nat. Nanotechnol 2015, 10, 1043–1047. 10.1038/nnano.2015.217. 26389661

[ref18] Miwa J. A.; Ulstrup S.; Sørensen S. G.; Dendzik M.; Čabo A. G.; Bianchi M.; Lauritsen J. V.; Hofmann P. Electronic structure of epitaxial single-layer MoS_2_. Phys. Rev. Lett 2015, 114, 046802 10.1103/PhysRevLett.114.046802. 25679902

[ref19] Zhang Y.; Ugeda M. M.; Jin C.; Shi S.-F.; Bradley A. J.; Martín-Recio A.; Ryu H.; Kim J.; Tang S.; Kim Y.; et al. Electronic structure, surface doping, and optical response in epitaxial WSe_2_ thin films. Nano Lett 2016, 16, 2485–2491. 10.1021/acs.nanolett.6b00059. 26974978

[ref20] Kim B. S.; Rhim J.-W.; Kim B.; Kim C.; Park S. R. Determination of the band parameters of bulk 2H-MX_2_ (M= Mo, W; X= S, Se) by angle-resolved photoemission spectroscopy. Sci. Rep 2016, 6 (1), 36389 10.1038/srep36389. 27805019 PMC5090988

[ref21] Kim B. S.; Kyung W.; Seo J.; Kwon J.; Denlinger J.; Kim C.; Park S. Possible electric field induced indirect to direct band gap transition in MoSe_2_. Sci. Rep 2017, 7 (1), 5206 10.1038/s41598-017-05613-5. 28701785 PMC5507882

[ref22] Kang M.; Kim B.; Ryu S. H.; Jung S. W.; Kim J.; Moreschini L.; Jozwiak C.; Rotenberg E.; Bostwick A.; Kim K. S. Universal mechanism of band-gap engineering in transition-metal dichalcogenides. Nano Lett 2017, 17, 1610–1615. 10.1021/acs.nanolett.6b04775. 28118710

[ref23] Han T.; Chen L.; Cai C.; Wang Z.; Wang Y.; Xin Z.; Zhang Y. Metal-Insulator Transition and Emergent Gapped Phase in the Surface-Doped 2D Semiconductor 2H-MoTe_2_. Phys. Rev. Lett 2021, 126, 106602 10.1103/PhysRevLett.126.106602. 33784141

[ref24] Brumme T.; Calandra M.; Mauri F. First-principles theory of field-effect doping in transition-metal dichalcogenides: Structural properties, electronic structure, Hall coefficient, and electrical conductivity. Phys. Rev. B 2015, 91, 155436 10.1103/PhysRevB.91.155436.

[ref25] Clark O. J.; Mazzola F.; Feng J.; Sunko V.; Marković I.; Bawden L.; Kim T. K.; King P.; Bahramy M. S. Dual quantum confinement and anisotropic spin splitting in the multivalley semimetal PtSe_2_. Phys. Rev. B 2019, 99, 045438 10.1103/PhysRevB.99.045438.

[ref26] Boccuni A.; Peluzo B. M. T. C.; Bodo F.; Ambrogio G.; Maul J.; Mitoli D.; Vignale G.; Pittalis S.; Kraka E.; Desmarais J. K.; et al. Unveiling the Role of Spin Currents on the Giant Rashba Splitting in Single-Layer WSe2. J. Phys. Chem. Lett 2024, 15, 7442–7448. 10.1021/acs.jpclett.4c01607. 39008656

[ref27] Vom Felde A.; Fink J.; Büche T.; Scheerer B.; Nücker N. Plasmons in the heavy alkali metals: strong deviations from RPA. Europhys. Lett 1987, 4 (9), 1037 10.1209/0295-5075/4/9/014.

[ref28] Laturia A.; Van de Put M. L.; Vandenberghe W. G. Dielectric properties of hexagonal boron nitride and transition metal dichalcogenides: from monolayer to bulk. Npj 2D Mater. Appl 2018, 2 (1), 6 10.1038/s41699-018-0050-x.

[ref29] Riley J. M.; Caruso F.; Verdi C.; Duffy L.; Watson M. D.; Bawden L.; Volckaert K.; van der Laan G.; Hesjedal T.; Hoesch M.; Giustino F.; et al. Crossover from lattice to plasmonic polarons of a spin-polarised electron gas in ferromagnetic EuO. Nat. Commun 2018, 9 (1), 2305 10.1038/s41467-018-04749-w. 29899336 PMC5998015

[ref30] Ma X.; Cheng Z.; Tian M.; Liu X.; Cui X.; Huang Y.; Tan S.; Yang J.; Wang B. Formation of plasmonic polarons in highly electron-doped anatase TiO_2_. Nano Lett 2021, 21, 430–436. 10.1021/acs.nanolett.0c03802. 33290081

[ref31] Caruso F.; Amsalem P.; Ma J.; Aljarb A.; Schultz T.; Zacharias M.; Tung V.; Koch N.; Draxl C. Two-dimensional plasmonic polarons in *n*-doped monolayer MoS_2_. Phys. Rev. B 2021, 103, 205152 10.1103/PhysRevB.103.205152.

[ref32] Xiang M.; Ma X.; Gao C.; Guo Z.; Huang C.; Xing Y.; Tan S.; Zhao J.; Wang B.; Shao X. Revealing the Polaron State at the MoS_2_/TiO_2_ Interface. J. Phys. Chem. Lett 2023, 14, 3360–3367. 10.1021/acs.jpclett.2c03856. 36995045

[ref33] Emeis C.; Mahatha S. K.; Rohlf S.; Rossnagel K.; Caruso F. Plasmonic polarons induced by alkali-atom deposition in hafnium disulfide 1T-HfS_2_. Phys. Rev. B 2023, 108, 155149 10.1103/PhysRevB.108.155149.

[ref34] Ulstrup S.; Miwa J. A.; Jones A. J.; McCreary K. M.; Robinson J. T.; Jonker B. T.; Singh S.; Koch R. J.; Rotenberg E.; Bostwick A. Discovery of interlayer plasmon polaron in graphene/WS_2_ heterostructures. arXiv, 2023. 10.1038/s41467-024-48186-4 PMC11519396 38714749

[ref35] Shi W.; Ye J.; Zhang Y.; Suzuki R.; Yoshida M.; Miyazaki J.; Inoue N.; Saito Y.; Iwasa Y. Superconductivity series in transition metal dichalcogenides by ionic gating. Sci. Rep 2015, 5 (1), 12534 10.1038/srep12534. 26235962 PMC4522664

[ref36] Taniguchi K.; Matsumoto A.; Shimotani H.; Takagi H. *Electric-field-induced superconductivity at 9.4 K in a layered transition metal disulphide MoS*_*2*_. Appl. Phys. Lett. 2012, 101, 042603 10.1063/1.4740268.

[ref37] Wu D.; Lin Y.; Xiong L.; Li J.; Luo T.; Chen D.; Zheng F. Enhanced superconductivity in bilayer PtTe_2_ by alkali-metal intercalations. Phys. Rev. B 2021, 103, 224502 10.1103/PhysRevB.103.224502.

[ref38] Saito Y.; Nakamura Y.; Bahramy M. S.; Kohama Y.; Ye J.; Kasahara Y.; Nakagawa Y.; Onga M.; Tokunaga M.; Nojima T.; et al. Superconductivity protected by spin–valley locking in ion-gated MoS_2_. Nat. Phys 2016, 12, 144–149. 10.1038/nphys3580.

[ref39] Zhou B. T.; Yuan N. F. Q.; Jiang H.-L.; Law K. T. Ising superconductivity and Majorana fermions in transition-metal dichalcogenides. Phys. Rev. B 2016, 93 (18), 180501 10.1103/PhysRevB.93.180501.

[ref40] Ilić S.; Meyer J. S.; Houzet M. Enhancement of the Upper Critical Field in Disordered Transition Metal Dichalcogenide Monolayers. Phys. Rev. Lett 2017, 119, 117001 10.1103/PhysRevLett.119.117001. 28949202

[ref41] Wang C.; Lian B.; Guo X.; Mao J.; Zhang Z.; Zhang D.; Gu B.-L.; Xu Y.; Duan W. Type-II Ising superconductivity in two-dimensional materials with spin-orbit coupling. Phys. Rev. Lett 2019, 123, 126402 10.1103/PhysRevLett.123.126402. 31633945

[ref42] Liu H.; Liu H.; Zhang D.; Xie X. C. Microscopic theory of in-plane critical field in two-dimensional Ising superconducting systems. Phys. Rev. B 2020, 102, 174510 10.1103/PhysRevB.102.174510.

[ref43] Wickramaratne D.; Khmelevskyi S.; Agterberg D. F.; Mazin I. I. Ising Superconductivity and Magnetism in NbSe_2_. Phys. Rev. X 2020, 10, 041003 10.1103/PhysRevX.10.041003.

[ref44] Semenov A. G. Pairing and Collective Excitations in Ising Superconductors. JETP Lett 2024, 119, 46–52. 10.1134/S0021364023603810.

[ref45] Zhao P.; Yu J.; Zhong H.; Rösner M.; Katsnelson M. I.; Yuan S. Electronic and optical properties of transition metal dichalcogenides under symmetric and asymmetric field-effect doping. New J. Phys 2020, 22, 083072 10.1088/1367-2630/aba8d2.

[ref46] Covaci L.; Berciu M. Polaron formation in the presence of Rashba spin-orbit coupling: implications for spintronics. Phys. Rev. Lett 2009, 102, 186403 10.1103/PhysRevLett.102.186403. 19518893

[ref47] Legma J.; Vacquier G.; Casalot A. Chemical vapour transport of molybdenum and tungsten diselenides by various transport agents. J. Cryst. Growth 1993, 130, 253–258. 10.1016/0022-0248(93)90859-U.

[ref48] Hoesch M.; Kim T.; Dudin P.; Wang H.; Scott S.; Harris P.; Patel S.; Matthews M.; Hawkins D.; Alcock S.; Richter T.; et al. A facility for the analysis of the electronic structures of solids and their surfaces by synchrotron radiation photoelectron spectroscopy. Rev. Sci. Instrum 2017, 88 (1), 013106 10.1063/1.4973562. 28147670

[ref49] Blaha P.; Schwarz K.; Tran F.; Laskowski R.; Madsen G. K. H.; Marks L. D. WIEN2k: An APW+ lo program for calculating the properties of solids. J. Chem. Phys 2020, 152 (7), 074101 10.1063/1.5143061. 32087668

