# Supplementary material for: Holstein Polarons, Rashba-Like Spin Splitting, and Ising Superconductivity in Electron-Doped MoSe2
Source: ACS Nano. 2024 Nov 26;18(49):33359–65. doi: 10.1021/acsnano.4c07805 (PMC11636255; doi:10.1021/acsnano.4c07805)
Supplement: Supplementary file 1 — nn4c07805_si_001.pdf [file nn4c07805_si_001.pdf]

Holstein polarons, Rashba-like spin splitting and Ising superconductivity in electron-doped $MoSe_2$

Sung Won Jung,[*,†,‡] Matthew D. Watson,[†] Saumya Mukherjee,[†,¶] Daniil V. Evtushinsky,[§] Cephise Cacho,[†] Edoardo Martino,[§] Helmut Berger,[§] and Timur K. Kim[*,†]

†*Diamond Light Source, Harwell Science and Innovation Campus, Didcot, OX11 0DE, United Kingdom*

‡*Department of Physics and Research Institute of Molecular Alchemy, Gyeongsang National University, Jinju 52828, Republic of Korea*

¶*Van der Waals–Zeeman Institute, Institute of Physics, University of Amsterdam, 1098 XH, Amsterdam, Netherlands*

§*École Polytechnique Fédérale de Lausanne, CH-1015 Lausanne, Switzerland*

E-mail: sungwon.jung@gnu.ac.kr; timur.kim@diamond.ac.uk

Supporting Information

Density Functional Theory (DFT) calculations

Band structure DFT calculations including spin-orbit coupling show that bottom of the conduction band at Σ-valley is lower for bulk $MoSe_2$ then for bulk MoS_2 and lower then bottom of the conduction band at K-valley. This makes $MoSe_2$ a much better choice to study polaron formation at Σ-valley. Importantly, in case of broken inversion symmetry, due to electric field built up on the surface caused by the alkali metal dosing, one expect corresponding spin-splitting at Σ-valley, like in the case of monolayer $MoSe_2$.

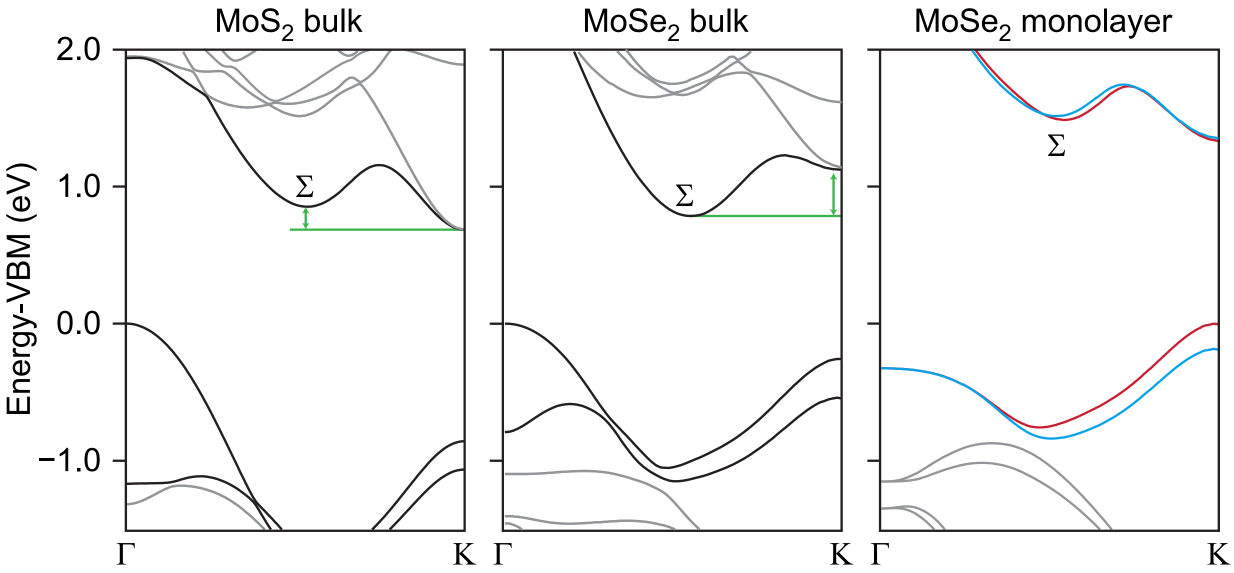

Figure S1: **DFT+SOC calculated electronic structure of bulk MoS_2, bulk $MoSe_2$ and monolayer $MoSe_2$.**

The evolution of $MoSe_2$ electronic structure with Rb dosing.

Photoemission data for conduction (Fig. S2) and valence (Fig. S3) bands together with corresponding Se 3d core levels data (Fig. S4) were measured for all Rb dosing sequences.

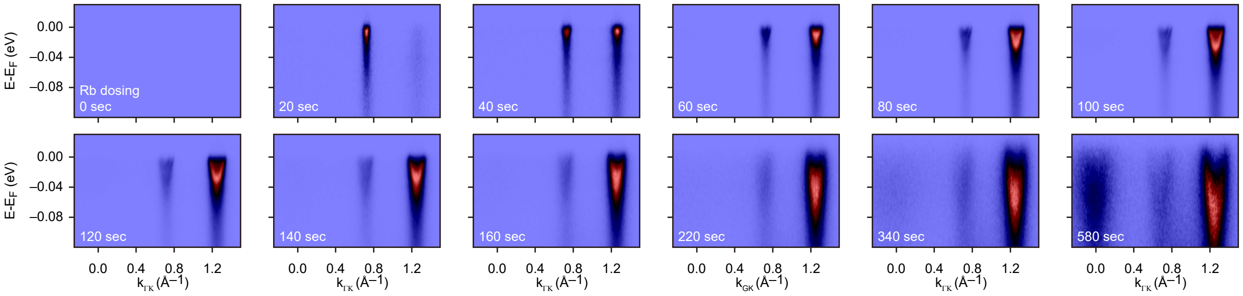

Figure S2: **Conduction band electronic structure at the Fermi-level of Rb dosed $MoSe_2$ surface.**

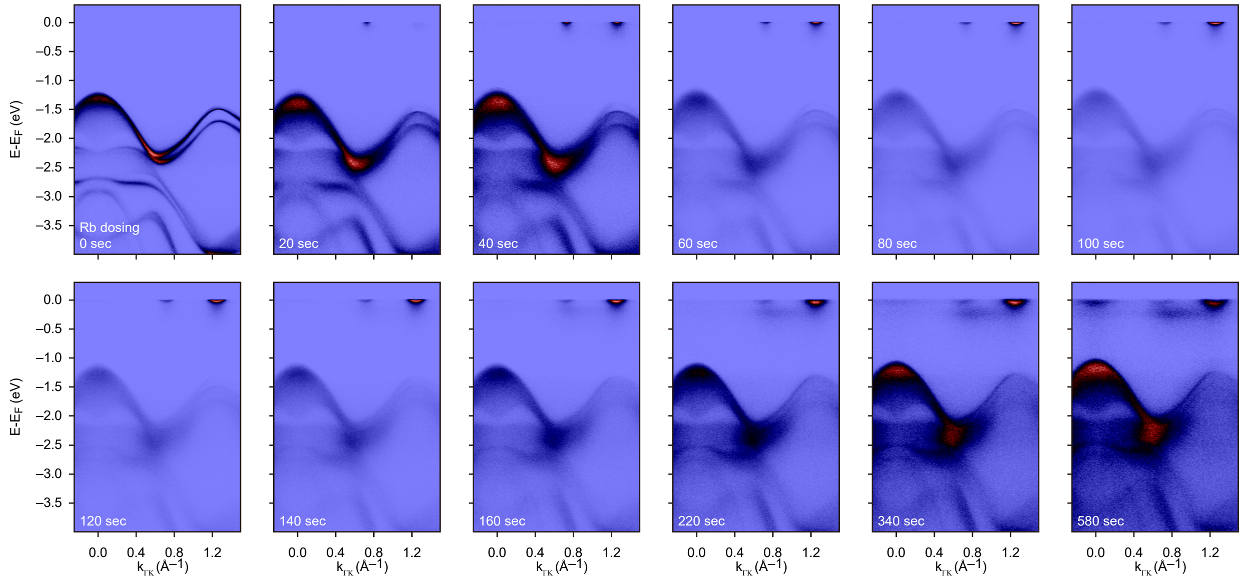

Figure S3: **Valence band electronic structure of Rb dosed $MoSe_2$ surface.**

A single $3d_{5/2}/3d_{3/2}$ doublet for a pristine $MoSe_2$ crystal splits into two components with Rb dosing as shown in Fig. S4(a). Similar to the case of $HfTe_2$[1] one could fit these core level data assuming bulk (P1) and surface (P2) components due to the electron doping of the $MoSe_2$ surface. Obtained peak area ratio and energy differences (or effective chemical shift) are shown in Fig. S4(b,c). Below critical dosing of 120 s one observes increase of the difference

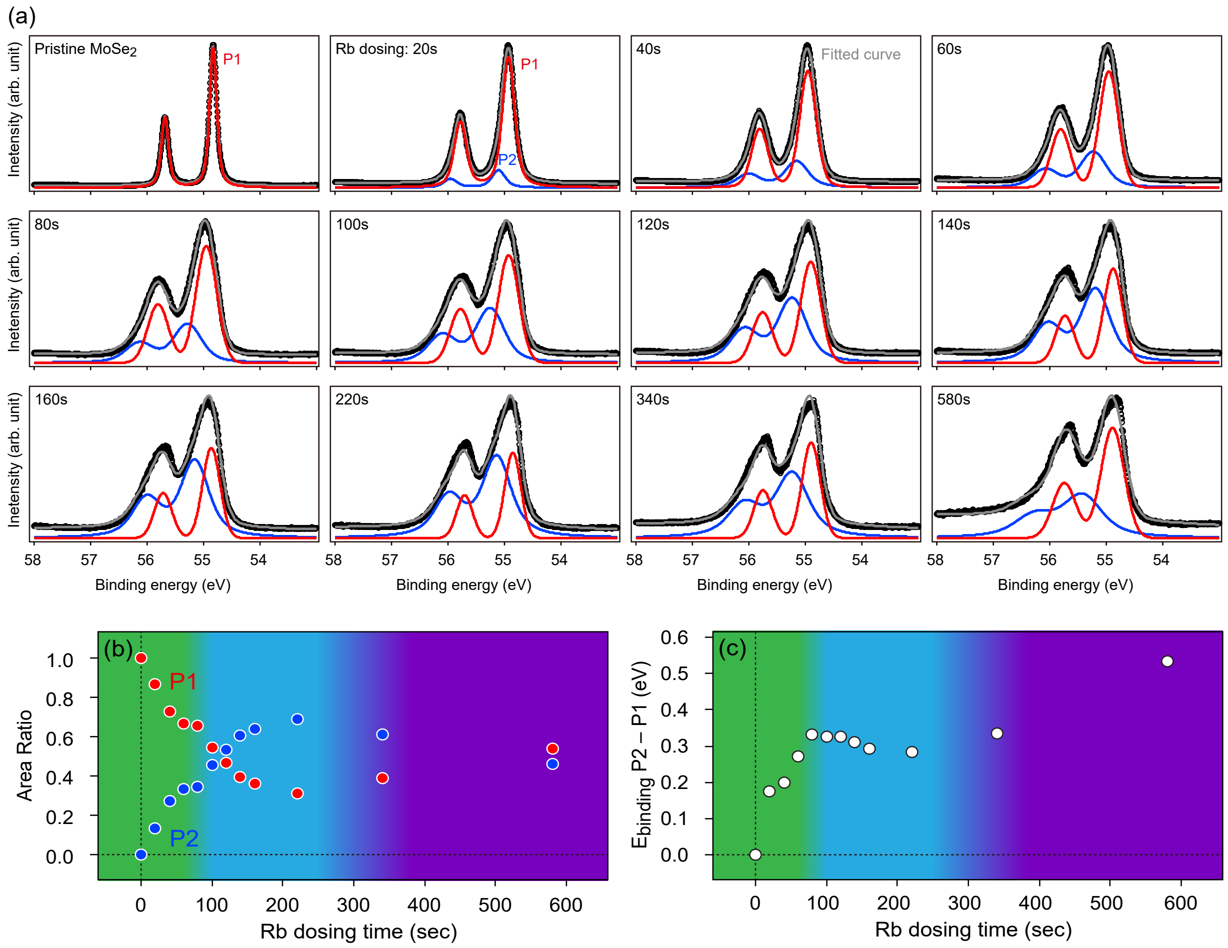

Figure S4: **Se 3d core-levels with Rb dosing.** (a) Se $3d_{5/2}/3d_{3/2}$ XPS data with Voight profile doublets fit for various dosing times. Red and blue curves indicate bulk (P1) and surface (P2) components; (b) Ratio of the bulk and surface components versus Rb dosing time; (c) Energy difference between bulk and surface components versus Rb dosing time; The background colour in (b) and (c) indicates surface dosing, intercalation and Rb metallisation regimes.

between bulk and surface doublets binding energies with dosing time. This corresponds to the gradual accumulation of Rb atoms, increased electron doping of the $MoSe_2$ surface, and corresponding increase of effective electric field. Beyond this critical dosing time, Rb atoms penetrate the van der Waals gap underneath the top layer, thereby physically and electronically isolating the topmost $MoSe_2$ layer. This clearly marks the second crossover from surface doping to the interlayer intercalation regime.

Doping evolution of the band gap in $MoSe_2$

Similar to the previously published ARPES data of alkali-dosed $MoSe_2$[2,3] we observe superposition of the bulk and surface electronic states. This is especially evident at top of valence band at K-valley where one detects weak surface related features at lower binding energies than bulk ones. At higher doping levels this might lead to a shift of the valence band maxima (VBM) from Γ to K valley and corresponding transition from indirect to direct band gap electronic structure. This has been clearly observed by ARPES in the case of $MoTe_2$.[3] However, in the case of $MoSe_2$ this is less obvious from the ARPES data.[2,3]

In Fig. S5(b,c) photoemission peak maxima of the conduction band for both Σ to K valleys shift towards higher binding energies, away from the Fermi level. However withing experimental broadening of corresponding spectra, one could not with absolute certainty conclude, that conduction band minima is shifting from Σ to K valley. Therefore, despite obvious spectral intensity shift from Σ to K valley in Fig. S2, we could not presume the crossover to a monolayer conduction band dispersion in alkali-metal dosed $MoSe_2$. Correspondingly, in Fig. S5(e,f) photoemission peak maxima of the valence band for both Γ to K valleys shift towards Fermi level with dosing. Withing experimental broadening of corresponding spectra, one could not determine, that valence band maxima is shifting from Γ to K valley. The lower intensity surface related features might be especially well masked by the bulk component at Γ valley as compared to K valley as seen in Fig. S7. Additional broadening of the spectra due to significant k_z dispersion at the centre of the Brillouin zone make distinguishing bulk and surface components there even more challenging. We can not exclude electron doping induced indirect to direct band gap transition in $MoSe_2$, however this might be beyond "surface doping" regime achievable with alkali metal dosing at the surface.

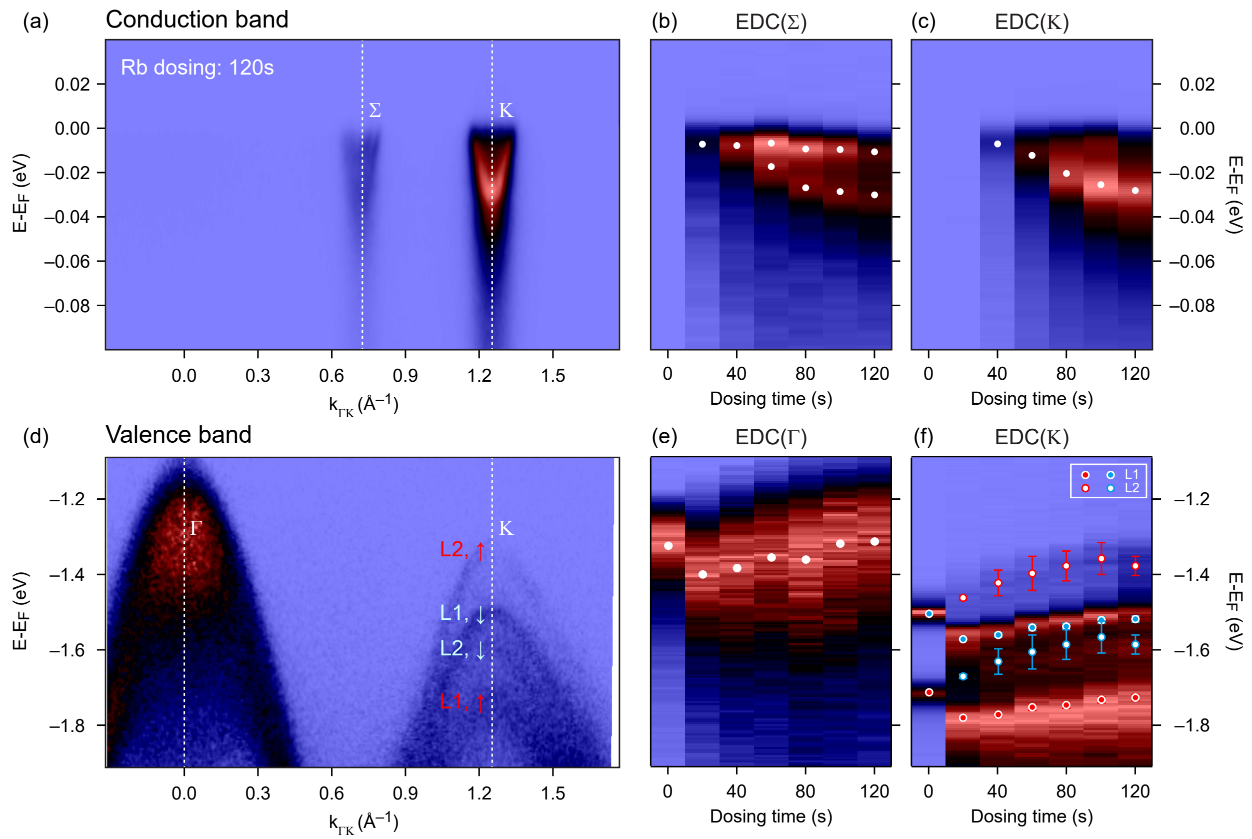

Figure S5: **Evolution of the conduction band minima and valence band maxima with Rb dosing:** (a) Conduction bands dispersion along Γ-K direction; (b) Dispersion of conduction band minima with surface doping obtained from EDC at Σ point; (c) Dispersion of conduction band minima with surface doping obtained from EDC at K point; (d) Valence bands dispersion along Γ-K direction. L1 and L2 notes topmost and second topmost layers, correspondingly; (e) Dispersion of Valence band maxima with surface doping obtained from EDC at Σ point; (f) Dispersion of Valence band maxima with surface doping obtained from EDC at K point;

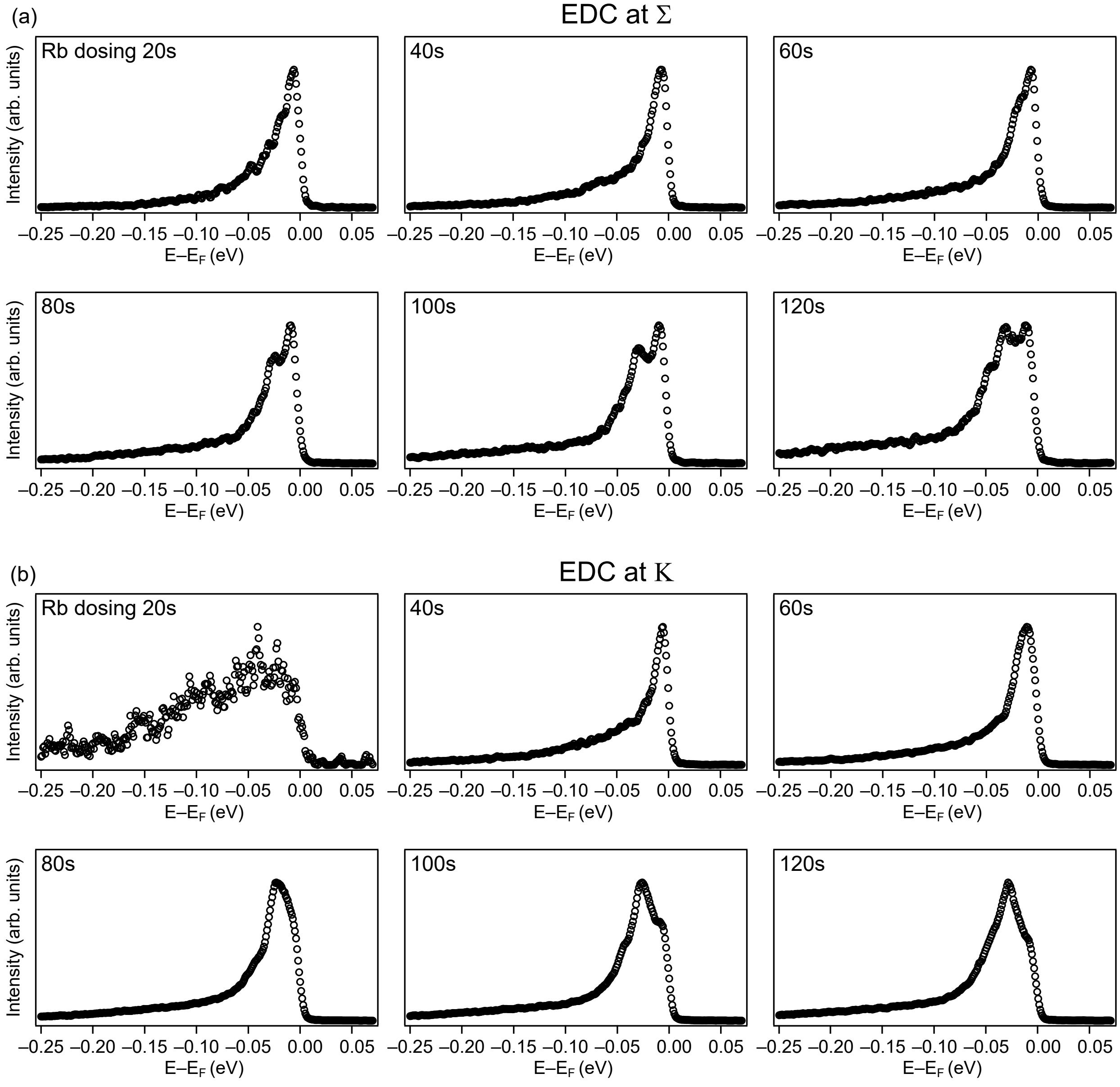

Figure S6: **Evolution of conduction band minima with Rb dosing:** (a) Energy distribution curves (EDCs) at Σ point; (b) Energy distribution curves (EDCs) at K point.

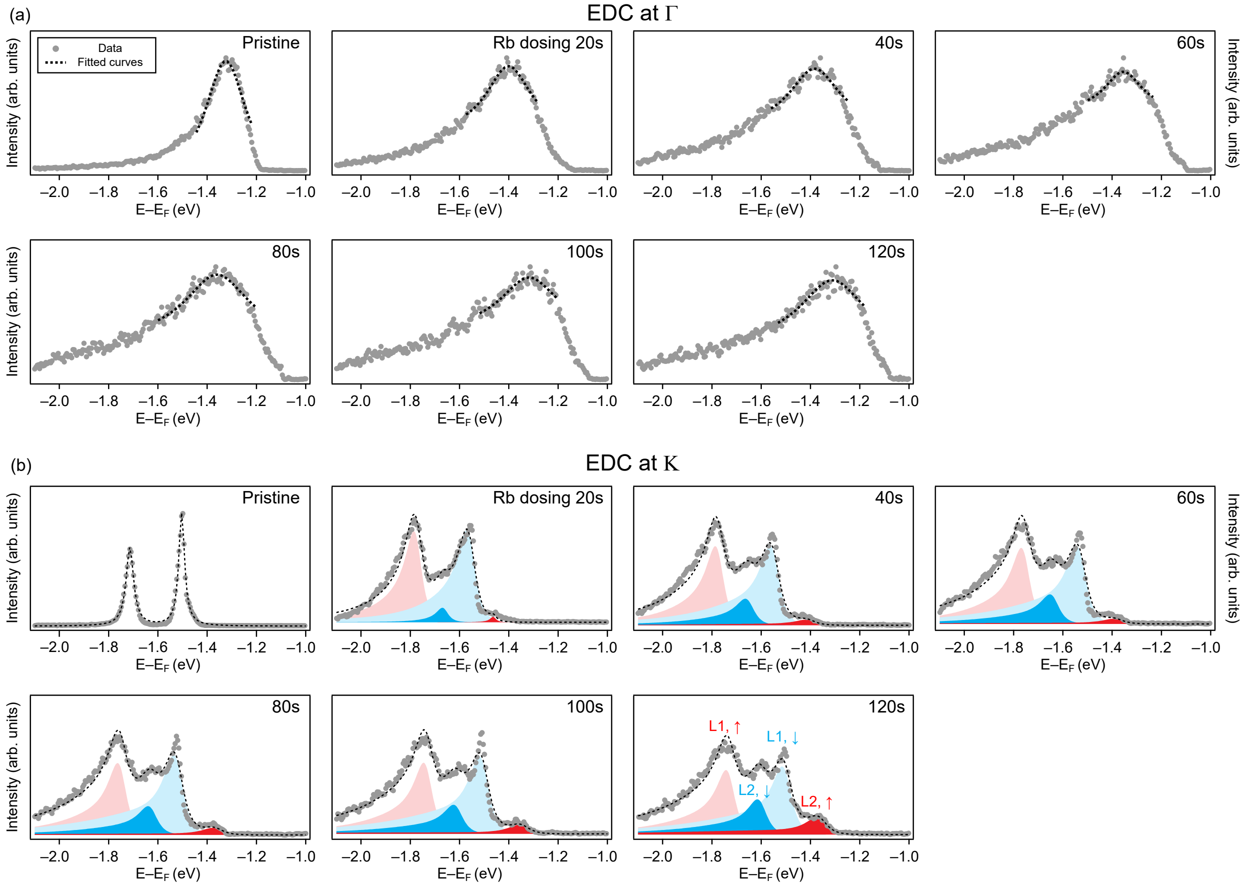

Figure S7: **Evolution of valence band maxima with Rb dosing:** (a) Energy distribution curves (EDCs) with fits at Γ point; (b) Energy distribution curves (EDCs) with fits at K point.

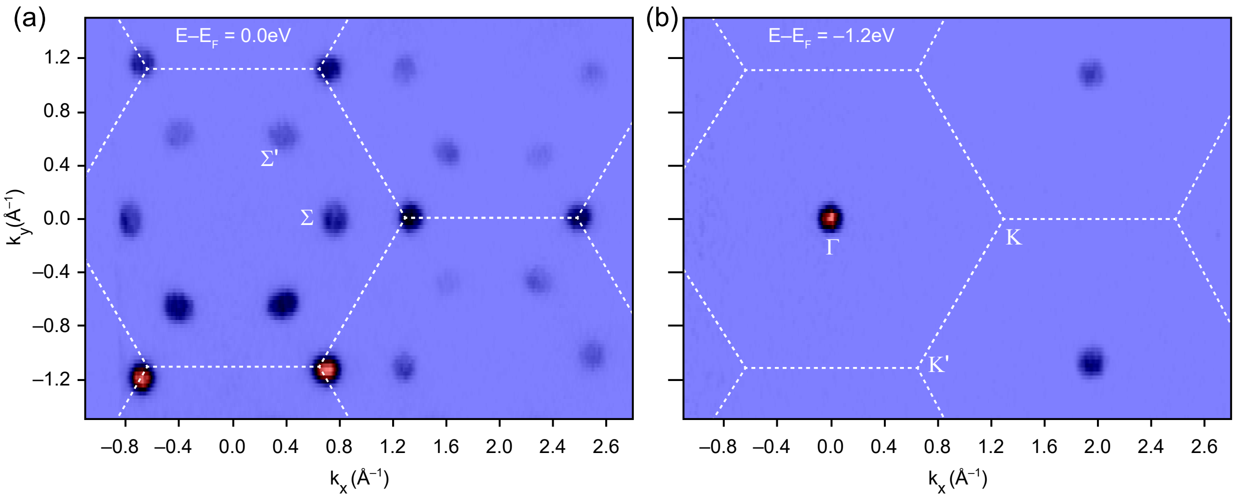

Figure S8: **Constant energy maps (hv=180eV) of $MoSe_2$ surface after 140 s Rb-dosing:**
(a) Momentum distribution map at Fermi level and (b) Momentum distribution map at the top of the valence band.

Rashba-like splitting at Σ valley

To analyse spin-splitting at Σ valley as a function of surface doping we used Rashba-like model with Γ as time-reversal invariant momenta (TRIM) point shown in Fig. S9(a). The

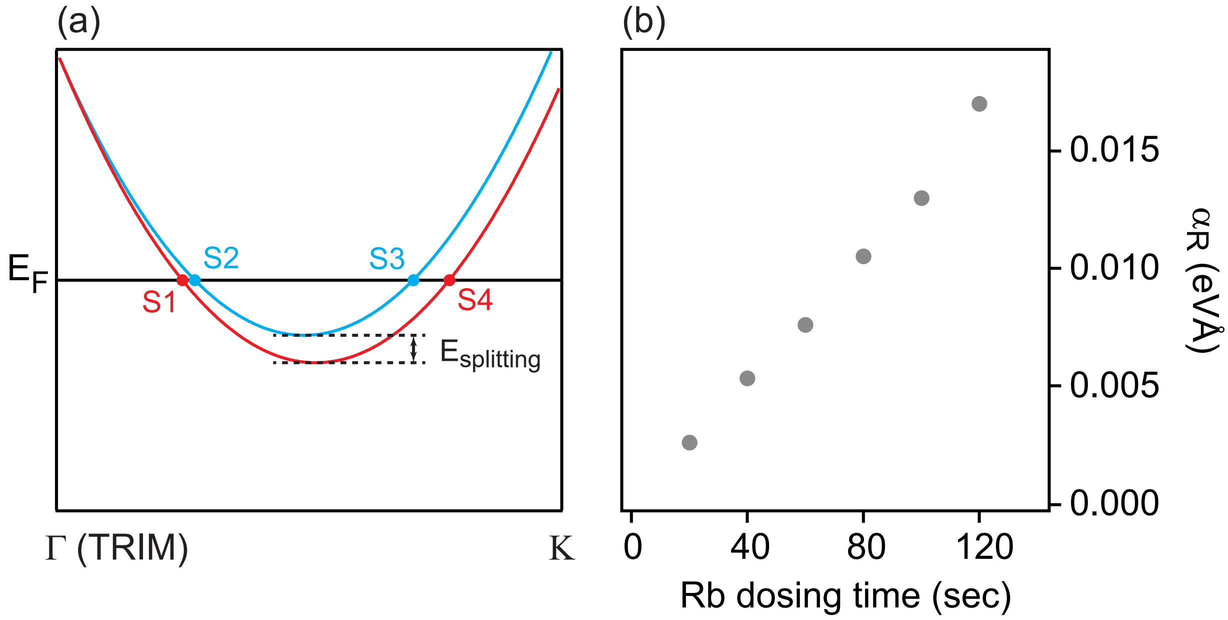

Figure S9: **Spin-splitting at Σ valley: (a) Rashba-like model with Γ as time-reversal invariant momentum (TRIM) point; (b) Extracted Rashba parameter (α_R).**

.

effective Hamiltonian can be written as:

$$H(\vec{k}) = \frac{\hbar^2}{2m^*}(\vec{k} - \vec{k_0})^2 \pm \alpha_R(\hat{n} \times \vec{\sigma}) \cdot \vec{k} + E_{offset} \quad (1)$$

where k_0 is the momentum of the conduction band minimum near Σ without spin-orbit coupling, σ is Pauli matrices, E_{offset} is the chemical shift due to the electron doping via Rb dosing and m^* is the effective mass.

From equation (1), one can make the system of equations with four k_F (S1,S2,S3 and S4)

shown in Fig. S9.

$$\frac{\hbar^2}{2m^*}(k_{S1} - k_0)^2 - \alpha_R k_{S1} + E_{offset} = 0 \tag{2}$$

$$\frac{\hbar^2}{2m^*}(k_{S2} - k_0)^2 + \alpha_R k_{S2} + E_{offset} = 0 \tag{3}$$

$$\frac{\hbar^2}{2m^*}(k_{S3} - k_0)^2 + \alpha_R k_{S3} + E_{offset} = 0 \tag{4}$$

$$\frac{\hbar^2}{2m^*}(k_{S4} - k_0)^2 - \alpha_R k_{S4} + E_{offset} = 0 \tag{5}$$

By subtracting the equation (5) with the equation (2), one can make

$$\frac{\hbar^2}{2m^*}(k_{S1} + k_{S4} - 2k_0) - \alpha_R = 0 \tag{6}$$

Likewise, one can subtract equation (4) from equation (3).

$$\frac{\hbar^2}{2m^*}(k_{S2} + k_{S3} - 2k_0) + \alpha_R = 0 \tag{7}$$

From equation (6) and (7), one can derive k_0 from four k_F values

$$k_0 = \frac{k_{S1} + k_{S2} + k_{S3} + k_{S4}}{4} \tag{8}$$

By replacing k_0 in equation (6) and (7), one can derive effective Rashba parameter α_R

$$\alpha_R = \frac{\hbar^2}{2m^*}\frac{(k_{S1} + k_{S4} - k_{S2} - k_{S3})}{2} \tag{9}$$

From equations (1), (8) and (9), the splitting energy $E_{splitting}$ can be derived as

$$E_{splitting} = 2\alpha_R k_0 - \frac{2m^*\alpha_R^2}{\hbar^2} \tag{10}$$

To fit the ARPES data, we used constant DFT value for the effective mass $m^* = 0.54m_e$

at Σ valley and k_F values obtained from MDC fits shown in Fig. S10. From these k_F values we estimate the experimental asymmetry of the Rashba splitting at Σ valley of about ~9%.

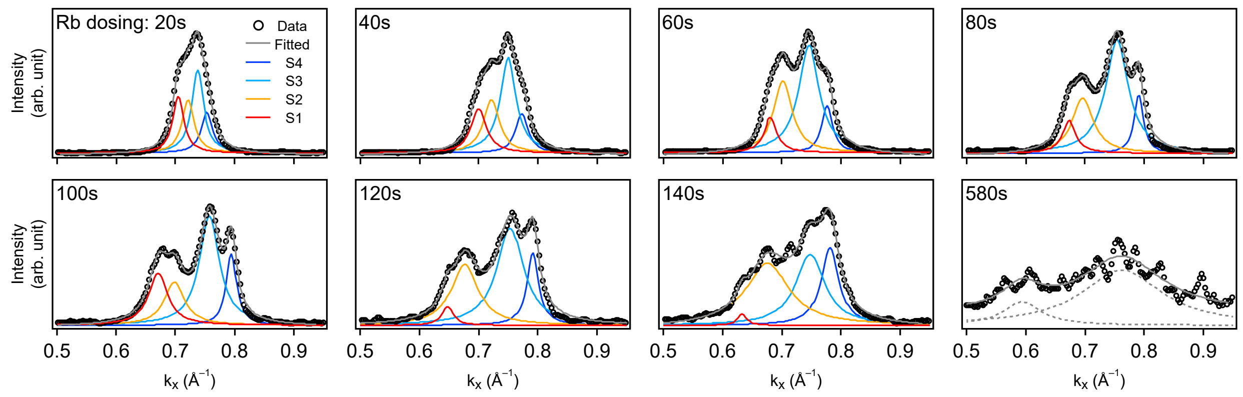

Figure S10: **Momentum distribution curves (MDCs) of Σ valleys at Fermi-level.**

The effective Rashba parameter α_R versus Rb dosing time is shown in Fig. S9(b). Corresponding extracted spin-split energy $E_{splitting}$ at Σ valley as a function of Rb dosing time is shown in the main text Fig. **??**.

For comparison, for Au(111) and Cu(111) surface states measured Rashba parameter α_R = 330 and 38 meVÅ give energy splitting $E_{splitting}$ = 110 and 16 meV, correspondingly.

References

(1) El Youbi, Z.; Jung, S. W.; Mukherjee, S.; Fanciulli, M.; Schusser, J.; Heckmann, O.; Richter, C.; Minár, J.; Hricovini, K.; Watson, M. D.; Cacho, C. Bulk and surface electronic states in the dosed semimetallic $HfTe_2$. *Physical Review B* **2020**, *101*, 235431.

(2) Kim, B. S.; Kyung, W.; Seo, J.; Kwon, J.; Denlinger, J.; Kim, C.; Park, S. Possible electric field induced indirect to direct band gap transition in $MoSe_2$. *Scientific Reports* **2017**, *7*, 5206.

(3) Kang, M.; Kim, B.; Ryu, S. H.; Jung, S. W.; Kim, J.; Moreschini, L.; Jozwiak, C.; Rotenberg, E.; Bostwick, A.; Kim, K. S. Universal mechanism of band-gap engineering in transition-metal dichalcogenides. *Nano Letters* **2017**, *17*, 1610–1615.
